# Human Split-Thickness Skin Allograft: Skin Substitute in the Treatment of Burn

**Published:** 2013-08-01

**Authors:** M. Mahdavi-Mazdeh, B. Nozary Heshmati, S. A. H. Tavakoli, M. Ayaz, F. Azmoudeh Ardalan, M. Momeni

**Affiliations:** 1 *Iranian Tissue Bank and Research Center, Tehran University of Medical Sciences, Tehran, Iran*; 2 *Shiraz Burn Research Center, Shiraz University of Medical Sciences, Shiraz, Iran*; 3 *Department of Pathology, Imam Khomeini Hospital, Tehran University of Medical Sciences, Tehran, Iran*; 4 *Motahhary Burn Research Center, Tehran University of Medical Sciences, Tehran, Iran*

**Keywords:** Lyophilization, Gamma irradiation, Skin allograft, Cytotoxicity, Tensile load, Skin burn

## Abstract

Background: Human skin allograft has been used as wound coverage for a long time; it is one of the most successful and widely used dressings for burn wounds in the world.

Objective: To prepare a freeze-dried human split-thickness skin allograft and evaluate its cytotoxicity, the structure and physical properties after processing methods and clinical efficacy in burn patients.

Methods: After ensuring tissue safety, we lyophilized human cadaveric partial thickness skin and exposed it to gamma radiation. Histopathological and immunohistochemical properties, tensile strength and *in vitro* cytotoxicity were assayed for the skin samples. Then, we tested the samples in 11 patients with deep skin burn.

Results: On histological and histopathological examinations, we found a normal skin structure. The tensile strength of the rehydrated freeze-dried human skin allograft was not lesser than the fresh human skin. Cell viability in MTT testing was more than 95%. None of our patients showed any signs of immunological reactions or complications.

Conclusion: Gamma-irradiated freeze-dried human split-thickness skin is safe and non-toxic and can be used for the treatment of patients with deep skin burn.

## INTRODUCTION

Burns, one of the most common causes of skin loss, may result in deep extensive skin wounds and even death. Therefore, finding an appropriate substitute for the skin in such patients is one of the main concerns of clinicians. An ideal skin substitute should be able to function similar to the natural skin with minimal immune reactions [1]. It should also be non-toxic and safe, easily handled, biocompatible, with no or low risk of disease transmission [2, 3]. An optimal wound dressing can restore the epidermal barrier function and will be incorporated into the healing process, relieves pain, protects the wound from infection and prevents fluid and electrolyte loss from the wound surface. Its long shelf life is an added value further to its low cost [3].

The best choice for burn wound dressing in full thickness injuries is split-thickness autologous skin graft [4-6]. Split-thickness skin autografts contain the epidermis and a variable thickness of the dermis, which saves time for healing of the remaining layers of the dermis at the wound site by secondary epithelialization and keratinocyte migration from its edges [7]. However, there are limitations in autologous skin usage, mainly due to pain and scar formation in donor sites. Furthermore, depending on the thickness of the harvesting sites of the skin, only three to four split-thickness skin harvests are possible from the same donor sites of the skin because of the delay in the re-epithelialization [8]. The last thing is that in extensive burn injuries, there is not enough autologous skin. We do not have extensive donor sites for recovery because of the limitation in the undamaged skin [3].

Whenever the provision of the autograft skin is limited, the mortality rate and hospital stay may increase. A suitable solution can be human skin allografts, as temporary dressing to prevent fluid and electrolytes imbalance and also microbial contamination.

Human skin allografts have been widely used as wound coverage for a long time and are one of the most successful and widely used dressings for burn wound worldwide [9-15]. They can improve and prepare the granulation tissue in the recipient bed. A human skin allograft becomes incorporated into the recipient tissue bed and will be covered by the recipient epidermal cells [1]. Human split-thickness skin allografts can be prepared and stored by various methods. One of the methods used is lyophilization. Dehydration due to lyophilization devitalizes most cells. Cell components will be destroyed and immunological reactions will be diminished as well. The “non-viable” skin allograft can be incorporated into the wound and make an appropriate dermal bed for further application of autologous skin grafts [3].

The objective of this study was to prepare a freeze-dried human split-thickness skin allograft and identify its structure and physical properties by histology, immunohistochemistry and biomechanics (tensile strength). We also tried to assess the *in vitro* biocompatibility of this skin allograft (cytotoxicity test) and then *in vivo* substitute in burn patients.

## MATERIAL AND METHODS

Donor screening, skin retrieval and processing

After screening age-limited cadaveric donors (1–60 years old) through medical history, physical examination and obtaining consent from their families, skin was retrieved within 15 hours of death using an electrical dermatome (Aesculap) with a cutting depth of 0.015 inches, according to standard protocols. The harvest was mainly from the lower limbs, back and buttocks in order to achieve both larger size tissue grafts and maintenance of the normal body appearance for the funeral. Skin strips were taken in sterile conditions and clean operating room after shaving and placed it in sterile containers of physiologic saline mixed with antibiotic. Afterward, multiple skin samples of 1×1 cm^2 ^were taken and placed in small containers of sterile normal saline for aerobic and anaerobic microbiological tests and also for mycobacterium assay and fungi cultures. Blood samples were also sent for culture and serological tests for antibodies against HIV type 1 and 2, human T-lymphotrophic virus type 1 and 2, hepatitis C virus, hepatitis B surface antigen and syphilis. All skin retrieval procedures were performed in a clean operating room. The skin containers were transported to tissue processing unit of Iranian Tissue Bank (ITB) in cold conditions (4 °C). After placing the skin in antibiotic and antifungal cocktail for 12 hours and ensuring tissue safety, hair follicles and hypodermal fat were removed and then the skin was rinsed with phosphate buffer saline (PBS) several times. Following draining, the partial-thickness skin was freeze-dried using a freeze dryer (Christ Alpha 2-4, Germany) until its water content became <5% (according to the standard protocol). The final step of sterilization was gamma irradiation with 25 kGy of packaged and labeled samples. All procedures were performed in clean room conditions.

Histopathological and immunohistochemical (IHC) examinations

After processing and before use, three samples of different lots of gamma-irradiated freeze-dried human split-thickness skin were randomly selected for histological and immunohistochemical analysis. Ten sections from each tissue block were prepared—two stained with hematoxylin and eosin (H&E) and eight with specific antibodies for the presence or localization of rabbit anti-mouse collagen IV, rabbit anti-mouse laminin, mouse anti-porcine vimentin and rabbit anti-chicken desmin in the processed skin. The slides were examined by an expert pathologist.

Tensile load testing

The tensile strength of the gamma-irradiated freeze-dried human split-thickness skin was measured by a tensiometer (Santam UTM). After 30 minutes of rehydration in sterile normal saline at room temperature, three skin sections measured almost 30×40 mm were considered for mechanical strength testing. Three similar samples of fresh human skin were used as controls. The graphs obtained by the computer program showed the relation between force and extension. The maximum extension of the rehydrated samples and fresh human skin before rupture and the force for rupture were recorded. The tensile strength was calculated using the following equation:

$\mathrm{Tensile strength}=\frac{\mathrm{Maximum tensiometer reading}(\mathrm{applied force})}{\mathrm{Cross sectional area}}$

Cytotoxicity assays

L929 mouse fibroblast cells prepared by Pasteur Institute of Iran were cultured in RPMI medium supplemented with 10% fetal calf serum (FCS), 100 Unit/mL penicillin and 100 μg/mL streptomycin. Cultures were maintained in air atmosphere containing 5% CO_2 _at 37 °C in an incubator for seven days. Cells that were passaged five or six times were used for cytotoxicity analysis. The cell suspensions were prepared enzymatically after detaching the cells by incubation in 0.05% (w/v) trypsin with 0.03% (w/v) ethylene-diamine-tetraacetic acid (EDTA) in Earl’s balanced salt solution from the tissue culture plates. Cells were seeded in 96-well microplates at a density of 1×104 cells/well and incubated for 24 hours to allow attachment. We used two pieces of rectangular 5×7-cm processed skin for preparation of extract according to ISO 10993-5 standard [16].

Cytotoxicity was assessed using the MTT (dimethylthiazol diphenyltetrazolium) assay. After addition of the extract solution to each well, they were incubated at 37 °C in air containing 5% CO_2 _and 95% relative humidity for four hours. Then, they were incubated for more than four hours in the dark at 37 °C in air containing 5% CO_2_ with addition of 100 μL of MTT 0.5 mg/mL solution. We also had two positive and negative control tests to compare and assess cell viability of the product. After incubation, MTT was aspirated and isopropanolol was added. The process was continued by agitating the plates until solubilization of formazan. Absorbance was measured at 545 nm, using a spectrophotometer. The mean test absorptions were calculated and assessed as percentage of controls and expressed as severe, moderate, mild or non-toxic corresponding to viability of less than 30%, between 30% and 60%, between 60% and 90%, and more than 90%, respectively.

Clinical application

After obtaining informed consent, we meshed the gamma-irradiated freeze-dried human split-thickness skin at 1–6 and 1–3 ratios and used the processed human skin in 11 patients with deep full thickness burn with more than 30% of their total body surface area (TBSA) affected; the participants had limited undamaged skin for complete autologous skin grafting. After excision of the burn sites, all 11 patients underwent partial (with autograft) to complete skin allograft meshed grafting. They were observed carefully for any reactions or complications until they were discharged from the hospital.

## RESULTS

Histopathological and immunohistochemical (IHC) examinations

Histological examination revealed an intact basement membrane. There was vacuolization of epithelial cells in epidermis. There were mild degenerative changes in dermal structures. On IHC examination, we found type IV collagen in the basal lamina of the epidermis and dermal vascular channels, as was expected. Laminin was not found. Vimentin staining revealed a scattered positive reaction in remnant cells of the dermis and the epidermis. Desmin was stained heavily in the basal layer of the epidermis and scattered smooth muscle bundles of the dermis.

Tensile load testing

The tensile strength of the rehydrated freeze-dried human split-thickness allograft was not less than the fresh human skin.

Cytotoxicity assay

**Figure 1:**
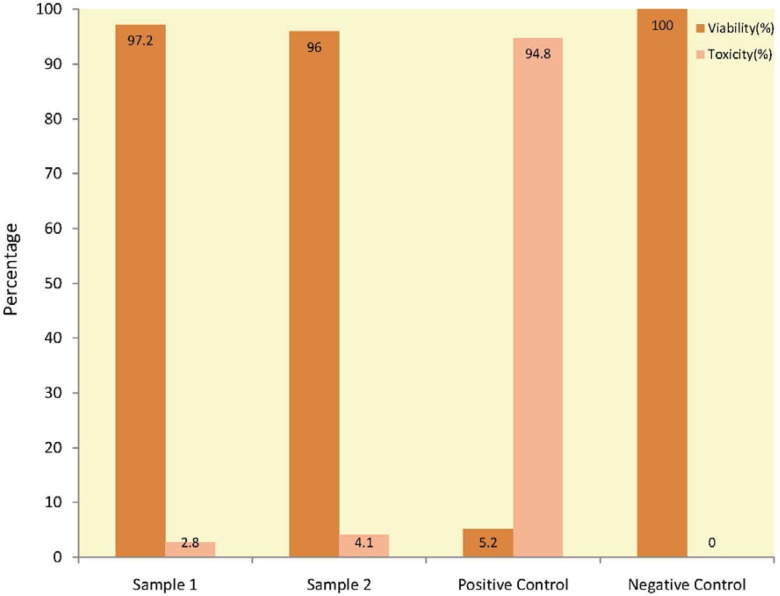
MTT staining results of freeze-dried human split-thickness skin allograft

Except for positive controls, none of our samples had signs of cytotoxicity. Cell viability was more than 90% in cultured L929 fibroblasts plates after MTT staining (Fig 1).

Clinical application

The mean±SD age of the 11 patients was 30.7±6.1 (range: 20–41) years; the male to female ratio was 6:5. The patients had a mean±SD burn surface area of 42.9%±16.6% (range: 17%–70%). The mean±SD time from burn injury to excision and allografting was 9.9±7.0 (range: 1–22) days. The mean±SD hospital stay was 32.2±26.5 (range: 9–96) days. The tissue adherence was good. There were no signs of infection, rejection or any other complications. Skin allograft detachment began 14 days after the grafting. Most patients were discharged with good functional results except for two who died of pulmonary emboli and sepsis—not due to an allograft reaction.

## DISCUSSION

An ideal skin substitute should function similar to the natural skin while inducing a minimal inflammatory response [1]. Shevchenko and MacNeil also emphasized that a skin substitute must not be immunogenic, toxic or induce excessive inflammation to be safe for patients [2, 3]. In our study, we found that our gamma-irradiated freeze-dried human split-thickness skin was safe and non-toxic while some cellular components were seen. We did not find any *in vitro* cytotoxicity and more than 95% cell viability was seen in MTT staining. This fact was in contrast with a study conducted by Dufrance, *et al*, who found that gamma-irradiation of non-decellularized human tissue (fascia lata) induced *in vitro* cytotoxicity [17].

In studies performed by Kearney, it was concluded that the gamma-irradiated freeze-dried split-thickness human skin was also comparable to the normal human skin because of its human source (cadaveric); he believed that lyophilization followed by gamma irradiation would remove the major antigenic parts of the harvested skin and the product can then be considered non-viable [18, 19]. Other studies also showed that freeze drying could reduce the immunogenicity of the skin [20] without interfering with its beneficial properties [21-24]. Our findings in the histological assay showed that some cellular components could be seen after freeze drying and gamma irradiation. Accordingly, the graft cannot be assumed non-viable. Similarly, Fawzi-Grancher reported that the freeze-dried human skin graft did not induce any harmful foreign body reactions after grafting [25]. We also found promising results in clinical evaluations in patients with deep skin burns. The patients did not show any complications or immunological reactions. The mortality rate was 19% within 32.2 days of their hospital stay, while Chua, *et al*, found a mortality rate of 16% within 48.3 days of hospital stay for early excision during the first 72 hours, and a rate of 45% within 58.5 days of hospital stay for grafting after 72 hours [26].

Although some studies have discussed the probability of weakening of the collagen matrix with gamma irradiation [27, 28], we did not find any significant difference in the mechanical strength between fresh and gamma-irradiated freeze-dried skin. In our clinical study, this characteristic helped surgeons to mesh at 1:6 and 1:3 ratios for covering more extensive burned areas on individual basis. Because of the skin allograft limitation, skin meshing may be a cost-effective procedure for covering more burn surface areas. Normal tensile strength is necessary for ideal skin meshing. Some strongly disagree with skin graft meshing as they believe it may postpone engraftment of the dermal elements. Obviously, it happen when irradiation dose is more than circa 25 kGy where it can lead to collagen denaturation [29] while our radiation dose was 25 kGy; therefore, it seems that the skin collagen fibers had not been denatured. Moreover, IHC showed a normal skin by identifying the basement membrane. Bourroul, *et al*, also reported the same results indicating no difference in the tensile strength between irradiated and non-irradiated skin allograft samples [30, 31].

One of the main concerns in selecting skin substitutes is the risk for transmission of diseases [3]. Because of the strict serological and microbial screening in our skin retrieval and processing protocols and use of gamma radiation for sterilization, the processed skin samples have low levels risk for transmission of diseases.

Another characteristic of our processed skin is its ease of use. It can be stored at the room temperature for up to two years because of the freeze drying procedure; in other forms of allograft skin samples, the shelf life is decreased because of cell viability. The gamma-irradiated freeze-dried human skin is easily handled, can be cut and formed in various sizes and shapes, can be meshed easily and the rehydration takes almost 30 minutes. These characteristics are of great advantage for a skin substitute [2, 3].

The main limitation of our study was that we did not biopsy the wound after allograft detachment to define if sloughing was only limited to epidermis.
